# How can aging be reversed? Exploring rejuvenation from a damage‐based perspective

**DOI:** 10.1002/ggn2.10025

**Published:** 2020-08-10

**Authors:** Bohan Zhang, Vadim N. Gladyshev

**Affiliations:** ^1^ Division of Genetics, Department of Medicine, Brigham and Women's Hospital Harvard Medical School Boston Massachusetts USA

**Keywords:** aging, biomarkers of aging, partial reprogramming, regeneration, rejuvenation

## Abstract

Advanced age is associated with accumulation of damage and other deleterious changes and a consequential systemic decline of function. This decline affects all organs and systems in an organism, leading to their inadaptability to the environment, and therefore is thought to be inevitable for humans and most animal species. However, in vitro and in vivo application of reprogramming strategies, which convert somatic cells to induced pluripotent stem cells, has demonstrated that the aged cells can be rejuvenated. Moreover, the data and theoretical considerations suggest that reversing the biological age of somatic cells (from old to young) and de‐differentiating somatic cells into stem cells represent two distinct processes that take place during rejuvenation, and thus they may be differently targeted. We advance a stemness‐function model to explain these data and discuss a possibility of rejuvenation from the perspective of damage accumulation. In turn, this suggests approaches to achieve rejuvenation of cells in vitro and in vivo.

## Graphical abstract

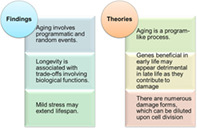

## 1 CURRENT UNDERSTANDING OF AGING

### 1.1 Aging as a combination of systematic transitions and random events

**FIGURE 1:**
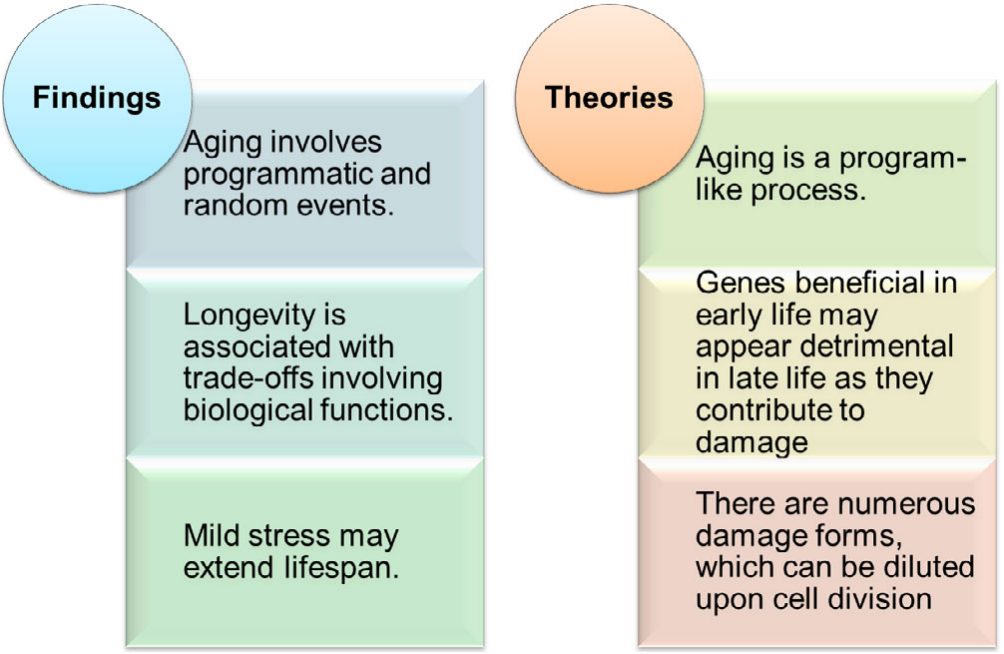
Facts and theories of aging. There are important experimental observations (facts) in the field of aging as well as many proposed theories to explain the aging process. They offer insights into particular aspects of the aging process, but require integration to better describe the complexity of aging

Aging is associated with an inevitable decline of organ and system functions, the cause of which remains a matter of debate. Although the possibility of lifespan extension by dietary, genetic and pharmacological interventions has been demonstrated for all common model organisms, indicating an association with slowing down aging, it has been less clear whether under certain circumstances aged organisms can be rejuvenated. Some researchers posit that aging is a programmed process, that the reverse‐program can be achieved, and some others believe that aging is mainly associated with random events, which cannot be reversed.^1^ Yet, these different views appear to point to particular features of aging, without emphasizing its multi‐dimensional nature. In fact, as we discuss below, emerging evidence suggests that aging is a combination of systematic and random changes (Figure 1).

One of the most obvious features of aging, besides its inevitability, is that aging is determined by numerous gradual changes that together relate to functional decline, including some that manifest as aging hallmarks.^2^ These changes follow certain age‐related trajectories, and although their origin is not well understood, they can be integrated into a set of biomarkers that track and characterize the process of aging. For example, on the phenotypic level, human facial phenotypes are known to gradually shift with age, which led to the development of biomarkers based on photographic images.^3^ This is also reflected in phenotypes such as cognitive deficiency.^4^ On the molecular level, the most precise biomarker of aging thus far is based on DNA methylation profiling and is known as the epigenetic clock.^5^, ^6^, ^7^ Different versions of this clock can estimate the biological or chronological age of humans and mice, thus distinguishing the rate of aging among different organs, and reporting the effects of longevity interventions.^5^, ^8^, ^9^ Importantly, interventions such as caloric restriction, rapamycin treatment, and certain gene perturbations can extend lifespan, regardless of differences between animals or even species.^10^, ^11^, ^12^ Moreover, age‐related events happen with surprising predictability across organisms and species, suggesting program‐like (programmatic, quasi‐programmed) features of aging.^13^, ^14^, ^15^, ^16^ However, aging is not a program like the developmental program of an organism, and it has no purpose. It is characterized by increasing randomness with time. For example, it has been observed through single‐cell RNA‐seq analyses that cell‐to‐cell heterogeneity increases with age, along with the perturbation of T‐cell activation that is associated with a decrease in T cell transcriptional variability.^17^ Variance in other biological parameters also changes with age, for example, DNA methylation and chromatin modifications.^18^, ^19^ Overall, aging can be described as a combination of predictable (program‐like) transitions and random events.

### 1.2 Longevity is associated with functional trade‐offs

It is important to distinguish the “rate of aging” and aging in general from longevity. First, aging is a process, whereas longevity is a quantitative feature of an aging organism. Secondly, aging may be understood by considering whether it applies to a living organism because not all organisms age, and why it happens. In contrast, longevity is best understood by asking questions about how long an organism lives and how this variable may be modified. Thirdly, longevity or lifespan is not only determined by the rate of aging, but also by lethal diseases and even conditions unrelated to aging but which may happen often in the old age. Such diseases, for example, lymphoma in lab mice may be targeted by certain interventions without affecting the rate of aging. Likewise, some diseases causing early death change mortality in early life without affecting aging. Aging is not necessarily accelerated in individuals with a markedly shorter lifespan or always happen proportionally to lifespan, although some short‐lived cases called progeroid syndromes do display certain features of accelerated aging. Although the nature of aging is not well understood, or at least there is still no consensus on its understanding, it is clear that it is possible to manipulate lifespan. Many interventions are now known that increase the lifespan of model organisms.^10^, ^11^, ^12^, ^20^, ^21^, ^22^, ^23^, ^24^, ^25^, ^26^, ^27^ In fact, increasing the lifespan of any model species is not at all unusual, as numerous conditions have been identified in large‐scale screens that lead to longevity.

As a rule, increased lifespan is associated with certain trade‐offs. It has been shown that there is a relationship between lifespan and factors that represent fitness and reproductive capacity. For example, inhibition of mTOR signaling promotes longevity, but suppresses the immune function.^28^ Growth hormone receptor (GHR) knockout mice have an extended lifespan, but the trade‐off is dwarfism.^10^, ^24^ Overexpression of telomerase can extend lifespan, but it is also associated with tumorigenesis.^29^, ^30^ The trade‐offs associated with longevity are also observed in the wild, as long‐lived species are often characterized by lower fecundity than short‐lived species^1^, ^31^. In other words, an intraspecific and interspecific extension of lifespan is usually accompanied by trade‐offs in “functions.” It is important to note that these functions usually benefit organisms when they are young. This relationship between longevity and fitness is also associated with the decline in the force of selection with age, which is widespread in nature with a few exceptions.^32^, ^33^, ^34^

Consideration of this aspect of aging leads to the concept of antagonistic pleiotropy (AP).^35^ This aging theory posits that there are genes and alleles with two‐sided effects that manifest differently with age, specifically being beneficial in the young (at or soon after the onset of reproduction) but deleterious in the older population. Because the benefits offered by such genes in early life can affect fitness, these genes are more likely to be selected, regardless of their negative effects in old age. This model explains the decline in the force of natural selection for aging population and is supported for example, by the effect of p53 gene on aging—it can protect against cancer‐related mortality, but can also impair normal tissue homeostasis and accelerate aging.^36^ On the other hand, the AP theory is less clear in addressing the “reverse trade‐off” in aging—hormesis^37^, ^38^. Hormesis refers to the observations that mildly harmful interventions often extend the lifespan of organisms. This effect is often explained by the fact that these manipulations activate stress response genes, benefiting animals throughout their life. However, if such genes follow the AP model, they should harm old animals and shorten lifespan, as is the normal case in high‐stress conditions when organisms exhibit distinct gene expression profiles.^26^, ^39^ Therefore, the AP concept, at least in its original form, is not an exhaustive description of aging, but rather a description of one aspect of aging. Although many theoretical models, such as AP, offer important insights into aging, they are incomplete in describing the complexity of aging. It is therefore important to unite these concepts into a single model, which not only defines the origin of aging and the effects of interventions on lifespan, but also explains approaches to rejuvenation.

## 2 AN INTEGRATIVE MODEL IN WHICH DAMAGE DRIVES AGING

### 2.1 Aging and entropy increase

**FIGURE 2:**
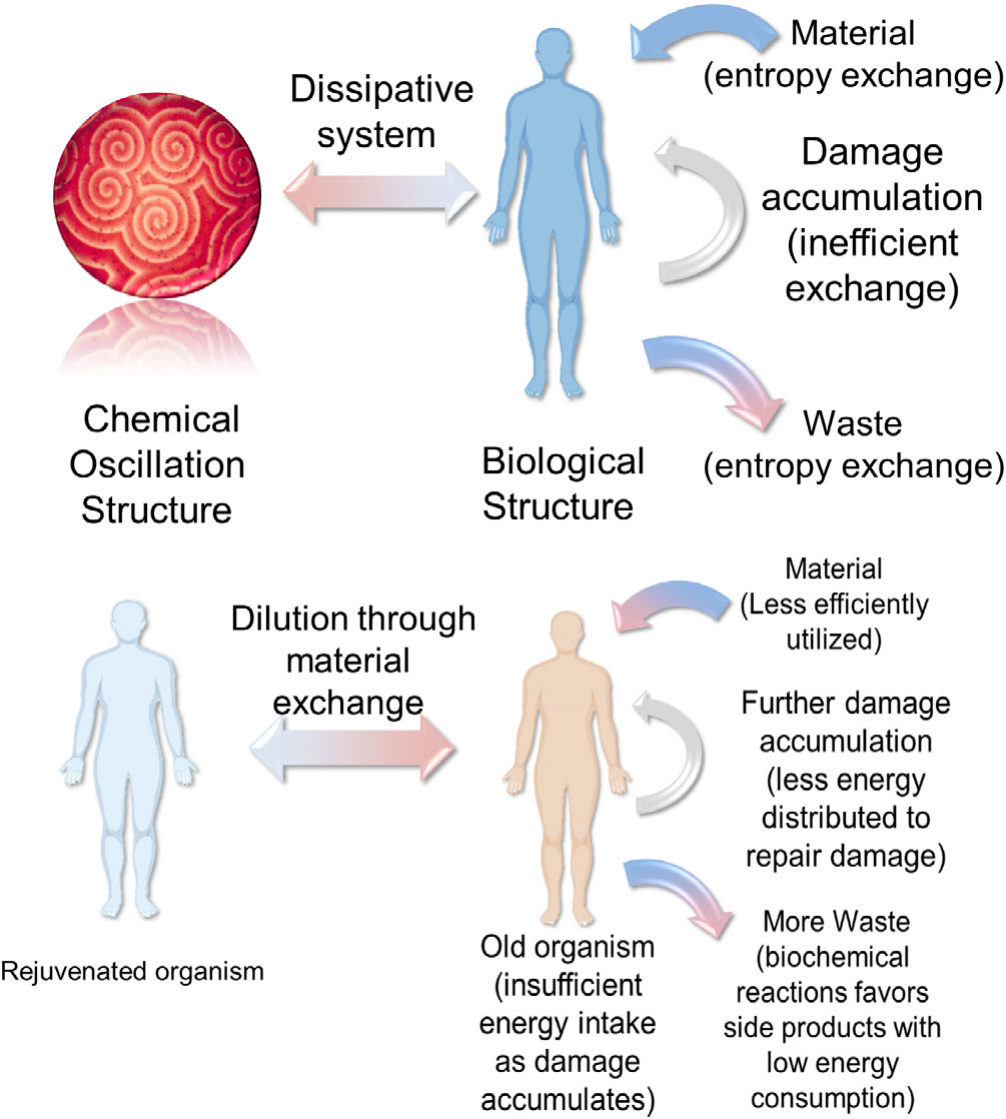
Aging from an entropy perspective. Upper: Analogy of aging to a chemical oscillation structure. Humans continuously exchange entropy with the environment by exchanging materials and energy in order to maintain the ordered biological structure. A restricted exchange of substances leads to aging. Lower: Further damage is caused by limits to obtain energy. The inability to take in substances disrupts functions and limits the ability to consume energy and repair damage, and the existing damage then makes biological systems produce more damage

Increased variation of different biological parameters during aging, as well as elevated Shannon information entropy (the degree of uncertainty or the amount of “surprise” in information such as those observed in DNA methylation profiles or gene expression patterns), suggests an analogy to the famous statement of the second law of thermodynamics: The total entropy or degree of disorder of an isolated system never decreases over time (Figure 2). This law stipulates that the order of energy flow favor the process that increases the degree of chaos. This analogy is consistent with the fact that Shannon entropy increases with age^24^, ^40^, ^41^. It is also interesting that the Horvath clock CpG sites exhibit higher Shannon entropy, providing insights into the relationship between entropy and systemic transitions during aging.^42^ Of course, what is different between this age‐related entropy increase and the law of thermodynamics is that living organisms including humans are not an isolated system. It is an open system that exchanges substances and energy with the environment.

The aging organism may also be viewed as a system with chemical fluctuations caused by biological reactions in the organism. This notion takes the whole organism as collected microscopically ordered patterns resembling oscillating chemical reactions. In such reactions, the chemicals involved form a dissipative structure far from the equilibrium, which allowed the raw materials to be transformed to maintain as a certain self‐assembled biological structure that is ordered both spatially and temporally.^43^, ^44^, ^45^, ^46^ With aging, program‐like changes reflect the temporal order of this structure, while the spatial order is damped over time as fewer and fewer materials effectively join the reaction. Like oscillating chemical reactions, this chemical fluctuation model suggests that the biological system gets materials (a negative entropy flow) from the environment to maintain the spontaneous self‐assembly of biological structures.^47^ However, it is harder to maintain such structures when the biological organization of an organism is constrained by its own biology and the genome evolution needed to maximize fitness, and is further impacted by age‐related changes, diminishing the ability to effectively exchange and distribute substances and energy. Therefore, these spatially ordered biological structures collapse as the negative entropy flow cannot be effectively transferred from the environment into the organism. Because the entropy of the system tends to increase, the organism must exchange substances with the environment to battle the increasing degree of disorder of the biological system over time, and it is increasingly unable to do so.

### 2.2 Aging is caused by systematic damage

The increased entropy problem arises from the fact that molecular damage and other age‐related deleterious changes are not efficiently removed from the organism. Organisms are composed of systems with all sorts of biochemical reactions. From an organic chemistry perspective, no perfect reactions exist that generate only products and no side products^48^, ^49^. The same idea applies to living organisms: biochemical reactions generate deleterious side products resulting in abnormal functions, such as somatic DNA mutations, extraneous RNA splicing variants, protein misfolding, and various other types. Taking the glycolytic pathway as an example, its side‐products are pervasively produced by essentially every participating gene product, and therefore glycolysis requires a specialized maintenance system composed of additional enzymes that target major by‐products of the 10 glycolytic reactions.^50^ Even for such a highly conserved pathway, there might be a certain degree of randomness that may generate the molecular damage; however, the specific side products of glycolysis are always produced, suggesting that much of this metabolic damage is not random. Organisms have evolved numerous mechanisms to deal with this damage; however, the protection mechanisms seem to be always inferior to the variety of damage forms generated; in addition, these mechanisms may also be imperfect themselves and thus introduce other forms of damage. Moreover, damage to energy‐producing systems (eg, mitochondrial dysfunction) limits energy production by organisms, compromising the damage control processes that require energy, for example, DNA damage repair. In turn, the accumulation of damage impairs biological functions, causing the increasingly frequent breakdown of biological systems.^51^ It should be stressed that the diversity of damage is not limited to molecular damage but involves many levels of age‐related deleterious changes. For this reason, it is convenient to designate the sum of this damage as the deleteriome.^48^, ^49^

The diverse types of damage in the aging cells suggest that an isolated non‐dividing cell or an organism with such nonrenewable cells cannot deal with them all, so the damage accumulates. These damages affect their normal cellular function and may trigger immune responses, which can in turn cause malfunction at many levels. For example, a mutation will lead to the respective error in the transcript and then in the synthesized protein or impair its regulatory function or gene expression. This type of damage may be random. A similarly damaged protein form may also occur due to a random error during transcription, translation, or protein modification. Nevertheless, most of such damages are systematic because they reflect particular gene expression processes involving multiple intermediate products, or are being part of functional molecular complexes, thus having a genetic or a program‐like nature. This damage is not entirely random, as it is generated by particular processes, involving genes that are encoded in the genome. This hierarchical reaction of damage explains why certain age‐related changes are program‐like, while others emerge in a random manner.

**FIGURE 3:**
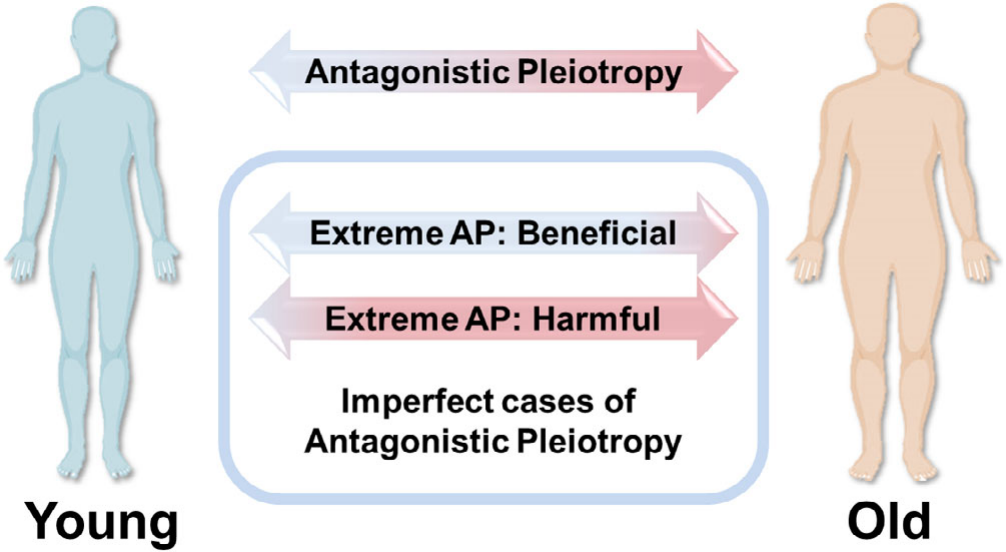
Antagonistic pleiotropy. The antagonistic pleiotropy (AP) theory states that certain alleles and genes that are beneficial in early life can be detrimental in later life, causing aging. However, the great majority of genes exhibit AP features, and this happens regardless of whether the organism containing these genes ages or not. We point out that the beneficial features of AP genes are represented by their functions, whereas the deleterious features are represented by damage generated because of these functions. This damage accumulates over time, leading to the appearance of the damaging effects of genes in late life. However, genes are not equal in their AP properties since in the extreme cases their beneficial effects may span most of the lifespan or be confined to a certain stage of development, and their deleterious effects may significantly contribute to the cumulative damage even in early life or contribute little over the entire life

Since genes are not perfectly precise in performing their functions, they will make mistakes that may cause damage as a result of these functions. In addition, a protein can perform different functions temporally and spatially, depending on the specific protein complex involving it.^52^ Therefore, all genes are AP‐like genes, or more broadly speaking, all biological products, and functions are antagonistically pleiotropic with regard to their functions and contribution to cumulative damage. Yet, judging by the normal function, not all genes are equal in the contribution of their antagonistic features. We may expect at least four distinct cases (described in Table 1): first, the AP genes that increase fitness significantly, whereas their resulting deleterious contribution is relatively small. These genes are highly beneficial in early life, but do not produce much damage over the lifetime, or organisms may die before the occurrence of the negative action of these genes. Certain genes related to age‐related diseases may be characterized into this category, as most organisms bear high mortality in the wild and cannot live up to the age when the negative effect of these genes takes place. Second, the AP genes that provide little benefit in early life, but significantly contribute to cumulative damage (Figure 3 **)** corresponding to the restricted case represented by another aging theory—mutation accumulation.^70^ Third, we predict inducible genes that cope with damage most effectively in later life when the damage they target most strongly affects function, thereby increasing lifespan. Such genes function in the maintenance and protection, via tumor suppression, apoptosis and senescent cell removal, and in some cases, autophagy.^55^, ^71^, ^72^ The fourth type of the AP genes comprises those that are essential during a certain stage of development but are damaging during other developmental stages and even after completion of development when the organism is supposed to have maximal fitness. Overall, it is clear that AP properties penetrate all of the biology but come in a variety of mechanistically distinct forms. In a way, the beneficial aspects of AP are the essence of life, whereas each deleterious property of AP is a side product or a consequence of life, and together these properties define the essence of aging.

**TABLE 1:** Examples of antagonistically pleiotropic features of genes and processes

Examples	Beneficial features	Deleterious features	Reference
Oncogene MYC WNT1	Cell proliferation stem cell maintenance	Tumorigenesis	53, 54
Tumor suppressor gene TP53	Inhibition cell proliferation of damaged cells	Apoptotic cell death necrotic cell death accelerated aging	55, 56, 57, 58, 59, 60
Cellular senescence	Prevents cell proliferation of damaged cells	Loss of cell function inflammation promotes tumorigenesis	61, 62, 63, 64
Immune response	Remove pathogens kill damaged cells kill neoplastic cells	Inflammation (inflammaging)	65, 66
Mild stress	Lifespan extension	Death frailty	38
Glyceraldehyde‐3‐phosphate dehydrogenase	Conversion of glyceraldehyde‐3‐phosphate to 1,3‐bisphosphoglycerate (glycolysis)	Side products: 1,4‐bisphospho‐erythronate, 4‐phospho‐erythronate, NADPHX, NADHX	50
Molecular oxygen	Aerobic respiration energy derivation	Intrinsic apoptotic signaling pathway in response to oxidative stress	67, 68, 69

### 2.3 Explaining rejuvenation from the damage dilution perspective in a stemness‐function model

Aging is an irreversible process, and most organisms can never escape the diversity and accumulation of damage that their own functions generate. To reduce damage, species with a simple organization may opt to discard some damage with a part of the cytoplasm, but this mechanism needs to be investigated in more complex species.^73^ interventions such as parabiosis may partially restore aged organ functions through transfusion of young blood to an old organism.^74^ This may be considered as a damage dilution process, where the old blood is diluted by the less damaged young blood. It was shown that, following hematopoietic stem cell transfer, the blood of the recipient follows the epigenomic age of the donor, suggesting a possibility to consistently generate younger blood than the actual age of the organism, if the source of hematopoietic stem cells is a young donor.^75^ It is important to emphasize that the transition to a younger age, based on one or more tissues being younger than the rest and younger than the chronological age, does not necessarily mean a longer lifespan for the subject, particularly if the lifespan is limited by a particular dysfunction or disorder that causes death. Recent studies suggested a possibility that certain drugs may slow down, and even reverse the epigenomic age, as defined by epigenetic clock biomarkers.^76^ These and other developments brought to light the possibility of “rejuvenation,” and even the prospect of reversing the biological age of an organism from an old to a young state.

Although somatic aging appears at first sight irreversible, we cannot bypass the fact that it is successfully reset to zero from generation to generation, suggesting that, during germline development, embryonic development, or some other phases of life there is a process that rewinds the aging clock. Somatic cell nuclear transfer shows that this rewinding process can be also induced in differentiated cell nuclei, although the mechanism is unknown.^77^ As discussed above, aging is caused by the accumulation of damage. However, this damage does not typically pass to the next generation to accelerate aging in the following generations, ultimately leading to species extinction. Therefore, some mechanisms must operate in the process of germline production, development, and maintenance that reverses aging and might provide the clue for selection against, diluting or even erasing of such damage. These mechanisms of dilution are currently unclear, although evidence suggests that they may involve a combination of cell division, cell selection, epigenetic remodeling, and global activation of genes, especially those genes for controlling DNA damage^78^, ^79^. These mechanisms allow cells to dilute even the scarcest molecular species such as functionally abnormal RNA, proteins, harmful metabolites, and those that would not be sensed by a cell. Thus, a combination of cell growth, selection, and proliferation dilutes mild damage, in addition to the removal of damage through specialized detoxification, repair, excretion, preemption, and other approaches. These mechanisms together allow the cells to keep the damage in control.^80^

It should be noted that division and dilution are not necessarily related in the context of proliferation of differentiated somatic cells, as, unlike germ cells or stem cells, these cells may undergo senescence or tumor transformation when proliferating in culture^81^, ^82^. This suggests that there is a particular relationship between cell division and damage dilution, whose mechanism is not yet understood. We think that this relationship is reflected, for instance, in the differences between early embryonic and aged cells, partially due to their different differentiation states. The former may stay in quiescent stage to avoid further damage or proliferate to select the cells with less damage. Compared to adult cells, embryonic cells specifically experience two waves of global demethylation and re‐methylation, establishing the same DNA methylation pattern for every generation.^83^ These differences suggest a possibility that certain embryonic cells and somatic cells have different modes and rate of damage accumulation and dilution through proliferation. From the damage perspective, the proliferation of cells with more specialized functions bears higher damage, as more specialized molecules are produced, allowing more side‐products to be generated. Furthermore, adult stem cells may overcome the proliferation limit when exposed to a mixed pro‐stemness signal.^84^ This shows that the combined effect of niche pathways that promote the stemness of the adult stem cells may act similarly to reprogramming. Thus, the difference in the damage accumulation between somatic cells and stem cells may lie, at least in part, in the cell matrix environment in which cells reside. Moreover, the environment may undergo a transition to sacrifice stemness for specific biological functions.

**FIGURE 4:**
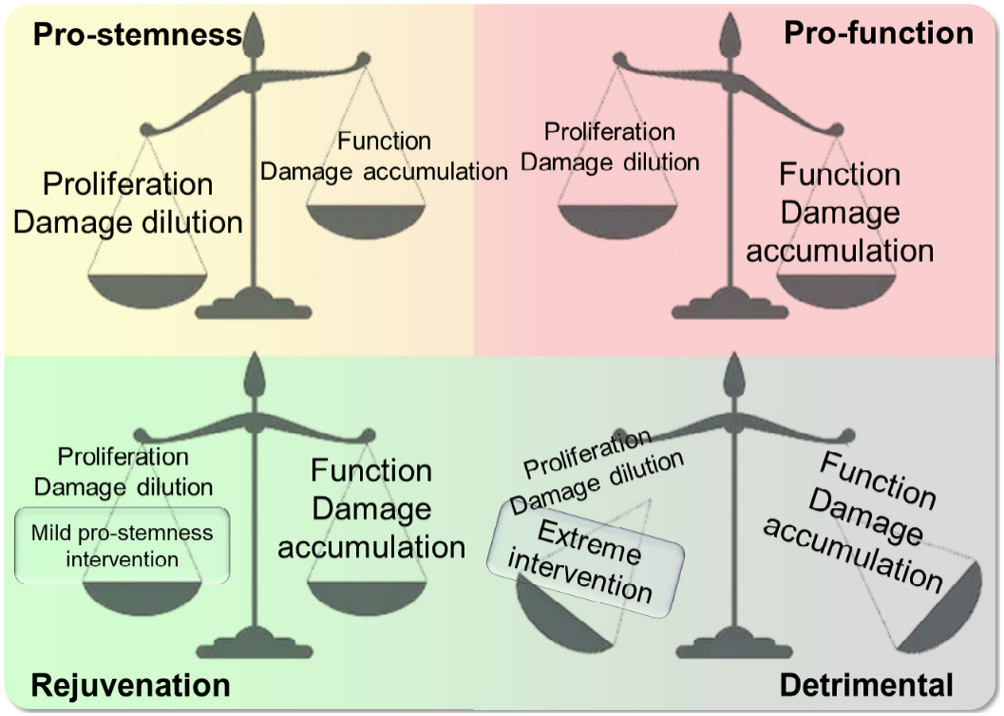
The stemness‐function model. This model posits the existence of two types of environment in an organism: the pro‐stemness state in the early life and regenerating cells, and probiological function in most tissues in the adult stage. Mild global activation of pro‐stemness genes in the pro‐function state may extend lifespan, whereas global overexpression of pro‐stemness genes may result in a detrimental effect

To visualize this stage‐shifting concept, we advance a weight‐scale metaphor, which we call a “stemness‐function” model (Figure 4 **and** Table 2). We designate the two states as “pro‐stemness” and “pro‐function” based on the balance between damage production and its removal by proliferation and apoptosis. During early life, organisms remain in a “pro‐stemness” state, encouraging cells to proliferate and grow so that the damage is unchecked and does not cause cell cycle arrest. In that state, although stem cells exhibit a limited intrinsic immune function, the function to recognize “self” and “nonself” is not yet fully developed, allowing a lower level of inflammation and an increased potential for regeneration.^97^ In contrast, in somatic cells, the damage generation can be sensed easier, triggering the reactions such as the DNA damage repair process, growth arrest, apoptosis, and immune responses. Therefore, organisms must undergo a transition from the “pro‐stemness” to “pro‐function” states, wherein differentiation and specification of cells are supported. Following this transition, the cells enhance their function in reproduction, damage sensing and apoptosis pathway, complete the immune function, and increase fitness by generating specific biological products related to their functions, while adult stem cells at this stage undergo gradual exhaustion^2^, ^98^, ^99^. At this stage, damage accumulation is spontaneous while damage dilution via proliferation is not supported in most cell types. During the process of fertilization or before/after it, this damage gets thoroughly checked, cleared and diluted by the transition to the “pro‐stemness” state^78^, ^79^, ^80^, ^100^. An example of such a “reset” function exists at fertilization in *C. elegans* where lysosomal functions in oocytes are enhanced by sperm‐secreted hormones, allowing degradation of protein aggregates and protein homeostasis.^101^ Such transitions are then followed by cell replication, allowing cells to enter the “pro‐stemness” state. Some cells may not enter this state successfully due to damage they bear, and these cells will be competed out by apoptosis, contributing to mortality in early life^102^, ^103^.

**TABLE 2:** Examples of age‐related manipulations following the stemness‐function model

Type	Manipulation	Outcome	Reference
Generation of iPSCs from somatic cells	Dilution of damage in pro‐stemness state	Age reset	5, 7, 8, 85, 86
Proliferation of somatic cells	Dilution of damage in pro‐functional state	Age acceleration, cellular senescence	6, 81, 82
Partial reprogramming	Mild pro‐stemness intervention	Potential age reset longer lifespan	87, 88, 89
Rapamycin	Potential mild pro‐stemness intervention	Potential age reset longer lifespan	90, 91
Hormesis mild activation of autophagy protein quality control tumor suppressor gene	Mild pro‐functional intervention	Potential longer lifespan	37, 38
Senolytics	Mild pro‐functional intervention	Potential longer lifespan	27, 72
Potent overexpression of iPSC reprogramming factors	Extreme pro‐stemness intervention	Loss of cell function tumorigenesis potential age reset	92, 93, 94
Forced proliferation of adult stem cells	Extreme pro‐functional intervention	Stem cell exhaustion age acceleration	95, 96

What perturbations might then be expected to delay or reverse aging? If a mild “pro‐function” feature is induced in the cells with the “pro‐stemness” state, it may extend lifespan as we learn from mild overexpression of certain tumor suppressors^104^, ^105^. Similarly, the weakened immune system upon rapamycin treatment provides an example that the opposite may also work^28^, ^90^, ^106^. On the other hand, if a specific function (supported by a certain gene) that shifts the system toward the “pro‐function” state is introduced, it may lead to death or premature aging, caused by a sudden increase in function and damage. This might be the case when tumor‐suppressor Tp53 is overexpressed in mice, and the animals show a significantly shorter lifespan.^56^ It should be noted, however, that similar cases of Tp53 overexpression in mouse models show an indistinguishable lifespan.^57^ Nevertheless, considering that cancer‐related deaths are more common in lab mice than in humans and that these risks are limited in these cancer‐resistant mouse models, there is still a possibility that the overexpression accelerates aging^57^, ^107^. Conversely, if a “pro‐stemness” signal introduced to cells in the “pro‐function” state, it may also cause deleterious effects, resulting in cell death or aberrant immortality. For instance, forcing cell proliferation by expressing oncogenes in fibroblasts promotes tumor transformation.^108^ A further support for this model is offered by the finding that human aging and cancer transformation exhibit transcriptional changes essentially in the opposite direction, along with the observation that cancer incidence in the elderly increases with age, together supporting the idea that cancer can be initiated by spontaneous processes that introduce aberrant “pro‐stemness” stimuli into the “pro‐function state”.^109^

## 3 APPROACHES TO REJUVENATION AND THEIR THEORETICAL BASIS

### 3.1 Rejuvenation through reprogramming

In our proposed stemness‐function model, the cell environment in the pro‐stemness state is different from that in the pro‐function state. In contrast to the rejuvenation of a whole organism, it should be easier to rejuvenate specific cell types in culture, because cultured cells are more homogeneous and less affected by extrinsic signaling crosstalk, hence more prone to go back to the pro‐stemness state. Supporting this notion is the induction of pluripotent stem (iPS) cells by Yamanaka and colleagues^85^, ^86^. Reprogramming adult somatic cells into iPS cells through the expression of four transcription factors (OSKM) achieves a cell fate like that of embryonic stem cells with the potential to develop as any part of the embryo. This can be interpreted as an example of damage dilution since the cells first undergo rapid proliferation along with the reduction of the size of each cell at the early state to become iPS cells, and iPS cells have a close‐to‐zero biological age as judged by the epigenetic clock biomarker^5^, ^8^, ^9^. These cells essentially revert from the “pro‐function” state to the “pro‐stemness” state and gradually reverse their age‐related damage, of which the aging biomarkers are the readouts.

However, as described before, forcing the expression of the stemness genes to overdo their effect on the “pro‐function” state may result in a detrimental consequence, such as weakened biological functions or tumorigenesis. One example is that promoting hematopoietic stem cell proliferation can lead to the accelerated aging potentially through exhausting stem cell.^95^ However, this may be different from treatments, such as young blood transfusion, which brings both the dilution of damage and a “pro‐stemness” general environment.

**FIGURE 5:**
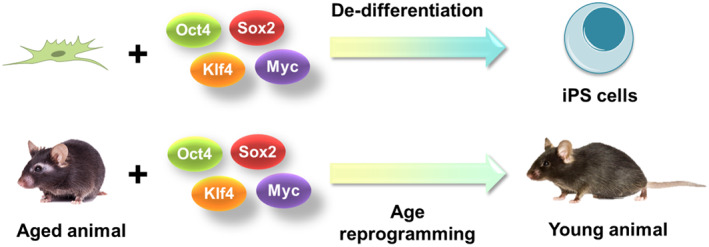
Two effects of in vivo reprogramming by Yamanaka (OSKM) factors are de‐differentiation and age reversal. De‐differentiation causes cells to go back to the stem cell lineage, whereas age reversal may lead to a younger biological age without de‐differentiation

It is important to note that even in cell culture, only a small portion of cells eventually become iPS cells. Reprogramming in vitro generates cell heterogeneity and most of the cells that undergo reprogramming differentiate into specific cell lineages, or transition through senescence, apoptosis, or premature differentiation.^110^ In addition, the generated iPS cells are more prone to tumor transition.^111^ This brings a major problem when researchers attempt rejuvenation using this approach in vivo. Such reprogramming in vivo in mice results in the loss of cell function and tumorigenesis^92^, ^93^. Therefore, mice constantly expressing OSKM die early rather than live longer. Later, researchers applied reprogramming with the premature withdrawal of the expression of OSKM to prevent tumor formation and loss of cell identity (Figure 5).^87^ Interestingly, an increased lifespan was observed in a fast‐aging mouse model, which may be explained by the partial transition from “pro‐function” to “pro‐stemness” states. In addition, a clear sign was found that the regenerative capacity increases upon injury, suggesting a transition to a more youthful state. Although an increase in lifespan has not been observed in normally aging mice, research suggests that this could eventually be achieved. A human in vitro reprogramming study based on the methylation clock biomarker found that there is a steady decrease in the methylation age upon reprogramming, as well as subsequent de‐differentiation characterized by the loss of molecular markers of cell commitment.^112^ We suggest an interpretation that the dilution of damage during the reprogramming results in the selection of a cell subpopulation that erases the age‐related methylation signature in the genome.

To move from partial reprogramming to true rejuvenation requires a mechanism to shift cells to a decreased age state without forcing them into a non‐physiological state of gene expression, or “confused” cell identity. This process might involve restoring original stem cell function through reprogramming differentiated cells to stem cells or the provision of specific signaling molecules that support stem cell functions by reprogramming differentiated cells in the stem cell niche. Recently, a study involving human muscle cells revealed a possibility that, by transiently expressing reprogramming factor (OSKM plus Lin28 and Nanog) mRNAs, one can restore young regenerative capacity and methylation age in human muscle stem cells without changing their identity, yet restore muscle physiological function of these muscle stem cells.^88^ This study, along with the previous work on partial reprogramming, suggests a possibility that there are indeed different states that represent age‐related transitions from those of developmental differentiation. If the gene sets contributing to age reversal can be distinguished from those involved in the reversal of differentiation state, rejuvenation may be accomplished more effectively in vivo. Considering that OSKM is not the only combination to induce reprogramming, and that some of them are predicted via omics methods and applied to trans‐differentiation, an interesting future approach may seek to identify the factors orthogonal to/downstream of OSKM that are only responsible for the age reversal and isolate their effects from the original reprogramming factors.^113^ As trans‐differentiation among adult cell types can also be achieved by inducing the expression of combinations of transcription factors, a similar strategy could be employed to find the genes of age reversal, skipping the requirement for pluripotency reprogramming but still erasing the cellular aging signatures.^114^

### 3.2 Rejuvenation by regeneration

The process of fibroblast reprogramming shares several interesting features with the wound healing process. Both involve massive cell senescence and cell death; both are related to cell de‐differentiation generating the cells that can grow into multiple cell types.^61^ Further, it has been shown that senescent cells promote both reprogramming and wound healing processes and that in vivo reprogramming promotes the regenerative capacity of animals, suggesting an intrinsic relationship between reprogramming and regeneration‐mediated wound healing.^61^, ^62^, ^115^ Thus, the possibility that regeneration can be harnessed to rejuvenate an entire organism should be taken into consideration and the differences between regeneration and rejuvenation should also be informative.

Animals have multiple ways to repair their wounds. The two main approaches are wound healing through regeneration and wound healing with scar tissues.^116^ When organisms employ the regeneration method, they de‐differentiate their tissues next to the wound to form a blastema. An almost identical tissue is then grown and differentiated from the blastema tissue. This process does not generate scars. Organisms such as axolotls, zebrafish, spiny mice, and even neonatal humans and mice can use this way to repair their wounded tissues.^117^, ^118^, ^119^, ^120^ Axolotls can repair their amputated limb and regenerate a completely new limb. They can also regenerate the cryo‐injured heart. Zebrafish can regenerate the tail if the wound is not severe. Neonatal mice can repair their skin without scar 3 to 5 days before birth, and they have the same heart regeneration capacity prior to being 7 days old.^121^ In contrast, adult humans and mice repair the majority of wounds with scar tissue.^117^ This process is triggered by growth factors produced by macrophages, which cause fibroblasts to proliferate and generate scar tissue to repair wounds.^122^ Although multiple biocompatible scaffolds have been applied as artificial and extracellular matrices to improve wound healing, there has not yet been any dramatic difference found through such biomaterials.^123^ Compared to the normal tissues, the scar tissues have collagen I aggregation and a different collagen structure.^124^ Notably, this scar‐inducing process does not restore the function of the originally wounded tissue. Therefore, the shift between regeneration and scar tissue formation suggests that the regenerative capacity of mammals decreases with age. Yet, it is unknown whether such a shift is merely due to the process of development or aging itself, as the question when the aging begins in early life is still not fully understood.

A recent study of deleterious somatic mutations and biomarkers of aging coupled with demographic analyses revealed that aging starts very early in life, whereas mortality is initially high and decreases in early life.^125^ Therefore, this shift in regenerative capacity may be due to the state of aging or the declining capacity to adapt to the changed environment. Weakened regenerative capacity in aged animals leads to impaired wound healing, especially in the skin and muscle^2^, ^126^, ^127^, ^128^, ^129^. It was suggested that this decline is closely related to the lowered immune function and inflammatory response during aging. Consistently, embryonic wounds have a low level of inflammatory cells and TGF‐beta 1 and 2 proteins, which might promote the regeneration process.^97^

This association between inflammatory responses and regeneration suggests that recognition and removal of non‐self and dying cells and cell debris might play a role in regeneration. A part of hydra can generate a new hydra if being pressed against the same part of another hydra, indicating that they do not distinguish self from non‐self.^130^ On the other hand, mammalian transplanted organs need treatment with immunosuppressors to keep their function. Depleting macrophages in humans and axolotls, or treating mice with the immunosuppressant, rapamycin can lead to impaired wound healing.^131^ Interestingly, besides inhibiting growth via mTOR function, rapamycin also downregulates TGF beta 1 and leads to a weaker immune function.^132^ This is consistent with our stemness‐function model: humans lose the regenerative capacity due to the transition to a “functional” state, and after this transition, the damage‐accumulating cells lead to aging and dying cells that increase inflammatory responses, exhaust stem cells, and accelerate aging. Rapamycin may partially reverse the organism to the “pro‐stemness” state by impairing certain functions including the immune response, thereby extending lifespan.

An important question is then unavoidable: If an organism has an unlimited regenerative capacity, will it have an unlimited lifespan? Hydra, planarians and some other species exhibit an almost unlimited capacity to regenerate with an exceptional lifespan^130^, ^133^. Axolotls show the ability to regenerate their limbs and heart, although it is not unlimited as axolotls fail to generate their limbs after multiple amputations.^134^ Humans and mice lost most of their regenerative capacity already during embryonic development, trading it for specific functions such as a tumor suppression.^95^ The application of partial reprogramming can now address this question; for example, the neurons responsible for the retina function are regenerated in the partially reprogrammed mice, challenging the dogma that neurons cannot be regenerated.^89^ This suggests another possibility, namely that partial reprogramming achieves its effect through a global increase of regenerative capacity. Such reprogramming can be accessed in vivo at several levels: by directing gene expression, changing the extracellular environment, and the exchange of signaling molecules, providing animals with a “pro‐stemness” environment.

**FIGURE 6:**
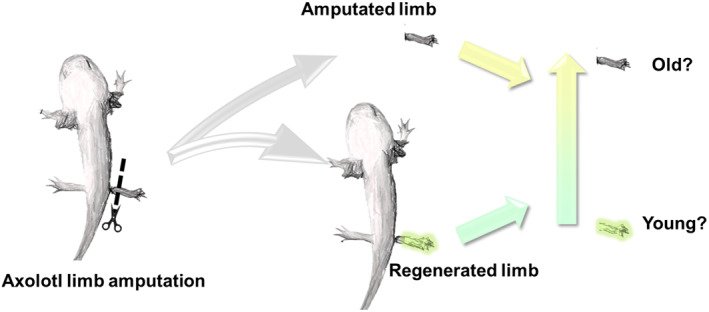
Assessing the biological age of regenerated tissues. Tissues undergo spontaneous de‐differentiation and re‐differentiation during wound healing by regeneration. Many features are shared between this process and reprogramming. It is possible that the regenerated tissues are younger than the original tissues based on their biological age. An example of axolotl limb regeneration is shown

The main question in the context of regeneration and aging is then whether the newly regenerated tissue is younger than the previously amputated tissue (Figure 6). Because regenerated tissues arise from de‐differentiated cells, in vitro cell reprogramming may be analogous to this process. It was shown that iPSC reprogramming erases epigenomic or transcriptomic features of aging from primary fibroblast cells and differentiate into the neurons with the same features, while directly converted neuron cells from primary fibroblast cells still retain age‐related transcriptome phenotypes.^135^ From this, we hypothesize that the regenerated tissues may be younger than the tissues they arise from. However, a more precise type of aging biomarker will be needed in the future to test this hypothesis. If the unlimited capacity to regenerate leads to a totally self‐renewable organism, a possible future approach for rejuvenation may be to identify the transition that allows humans to temporarily reverse to the “pro‐stemness” stage. With the transition to a state supporting unlimited regenerative capacity, one may be able to achieve an infinite self‐renewal of tissues with minimal loss of developmental identity or neoplastic transformation.

## AUTHOR CONTRIBUTIONS

**Bohan Zhang:** Conceptualization; visualization; writing‐original draft; writing‐review and editing. **Vadim Gladyshev:** Conceptualization; funding acquisition; supervision; writing‐review and editing.

## CONFLICT OF INTEREST

The authors declare they have no conflicts of interest.

[Correction added on 1 December 2020, after first online publication: Peer review history is not available for this article, so the peer review history statement has been removed.]

## Supporting information

Transparent‐Peer‐Review‐Record
