# Supplementary material for: How can aging be reversed? Exploring rejuvenation from a damage‐based perspective
Source: Adv Genet (Hoboken). 2020 Aug 10;1(1):e10025. doi: 10.1002/ggn2.10025 (PMC9744548; doi:10.1002/ggn2.10025)
Supplement: Supplementary file 1 — Transparent‐Peer‐Review‐Record [file GGN2-1-e10025-s001.pdf]

Advanced Genetics

How can aging be reversed? Exploring rejuvenation from a damage-based perspective

Bohan Zhang and Vadim N. Gladyshev*

*Corresponding author

Review timeline:

Date Submitted: 10/02/2019
Editorial Decision: Minor Revision 12/04/2020
Revision Received: 01/27/2020
Editorial Decision: Minor Revision 02/12/2020
Revision Received: 03/02/2020
Accepted: 03/17/2020

Editor: Alison Liu

1st Editorial decision	12/04/2020

Editorial Recommendation:
This is a thought-provoking review that discusses several fundamental questions in the aging biology, proposes a nice damage-based seesaw model, and suggests new directions to reverse the aging process. The authors point out the different mechanisms between de-differentiation and rejuvenation, and that different reversal strategies are needed to the cells at "pro-stem" and "pro-function" state. They introduce a unified aging model, attempting to explain the aging process at cellular, tissue, and organism levels. However, the model could be more carefully specified by adding examples of genes that promote stemness or (commitment, differentiation?) "function". Some of the roles of damage and niche signals in resetting age in stem cells and (their model) iPSC could be discussed. Reviewers are thoughtfully engaged and provide excellent and constructive comments. All referee comments are useful and should be addressed. As to that there was not enough critical thinking about the opposing views, we would not insist upon balance unless the piece is to be longer Review (which would probably increase its impact). The Perspective can well be one-sided, but not unskeptical. Please consider our comments on the specific referee points and additional recommendations:

(1) Please address the differences between aging and longivity

(2) **The genetic programing of aging:** Regarding if aging is a genetically programmed process, the authors concluded that it is "a combination of predictable transitions (program-like) and random events". The question is whether the different aging rates among tissues have distinct roles in the lifespan.

(3) **Adding a table of the AP genes** can help explain the tradeoff between the fitness in young lives and longevity or aging. This will make the paper more useful and increase its impact. Some of the best examples are in model organisms. Those that benefit the fitness seem to be driven by reproductive success. You might also want to explain the "deleterious" phenotypes caused by these genes in old organisms.

(4) **Stress vs. lifespan:** To be convincing, you might want to provide examples of harmful phenotypes imposed by mild stress that extend the lifespan. This will also help stress your point that this does not fit the old AP theory. High stress may increase the biological age and shorten the lifespan (doi: 10.1016/j.arr.2018.10.001), which fits the AP theory. It is possible that mild stress activates different sets of genes from high stress, and both have opposite effects on lifespan. For example, in plants, high, ambient, and cold temperatures can activate different sets of miRNAs and their targeting genes.

(5) **List some cases of genes on the seesaw model to explain dilution of damage.** According to the seesaw theory, the damage can be diluted during frequent proliferation in embryo and germ cells. What are the experimental supports for this? How does the dilution explain that the DNA repairing plays a pivotal role in

maintaining low mutation rate in germ and stem cells (doi: 10.1038/ncomms15183)? Are good cells selectively propagated over damaged cells due to cell cycle arrest? This raises interesting questions about whether and how damaged genomes provide a proliferative stimulus to stem cells and this could be a fascinating extension to the review – this needs to be explained using experiments. In addition, a study showed aging cells can simply dump the cytoplasm containing the damage components (doi: 10.1038/nature21362) - This might well be an alternative way of dilution.

Does the damage dilution help erase and reestablish DNA methylation during the embryonic reprogramming? In cancer cells, DNA damage and epigenomic landscape intertwines to promote cellular growth and proliferation (Please briefly comment on this, as this can be a major review).
It might also be useful to discuss the case of Dolly, the sheep, who have the DNA mutations (damage) in the pro-function state nucleus of mammary gland cell been diluted after being transferred to an unfertilized oocyte (nucleus was removed) and proliferating during embryonic development in the apparently pre-stemness environment.
(6) **Re-setting the aging clock:** Discuss briefly the embryonic reprogramming as re-setting the aging process and discuss the role of stem cell niches in providing the equivalent of reprogramming signals.
(7) **Parabiosis and classical "rejuvenation therapies"** are complex to interpret and should not just be labeled "regeneration". In terms of iPS reprogramming resets aging. Should there therefore be analysis or at least comment on the role of c-Myc?
(8) **The analogy between aging and entropy:** You might want to define several thermodynamics-related concepts to help the reader follow the discussion, for example, the law of thermodynamics, "increased variability during aging", "methylation entropy", "isolate system" vs. "open system". Please cite "What is life?" explaining why Schrödinger did not think that life followed the physical laws.

Please explain how the negative entropy flow happens during the aging process. On the top of the suggestion that the negative entropy flow was caused by the inhibition of energy exchange and distribution, could you also comment on how the reduction of NAD and energy decline (enthalpy, ΔH, reduced?) in aging cells contributes to the increase of entropy? Entropy is a measure of energy dispersal after all. According to $\Delta G = \Delta H - T\ \Delta G$, if Gibbs free energy, ΔG, is negative, then the reaction is spontaneous with increased entropy. The decline of energy exchange could result from reduced energy production. For example, DNA damage or somatic mutation accumulation can increase the genomic entropy, and this might be due to the energy decline, because repairing, removal of dying cells, and energy exchanges and distribution are all energy-driven processes. Heterochromatin loss and hypermethylation and activation of transposons, and increased DNA damages during the aging process and cellular enlargement are good examples to explain the increase of entropy during aging.
Schroedinger is relevant, but extended discussion of Shannon's idea of information and entropy on opposite sides of a possibly different seesaw would need some discussion of how Shannon's information and entropy can be measured in aging cells, for example (Slieker 2016 10.1186/s13059-016-1053-6 , Jenkinson 2017 10.1038/ng.3811). In this paper, https://doi.org/10.1186/s13059-019-1753-9, the authors showed the Horvath clock CpG methylation sites are of high entropy, or high variability, perhaps suggesting that some biological processes are specifically vulnerable for the changes during the aging process.
(9) Damages are caused by the side products of the reactions. In organisms, enzymatic reaction side products are often recycled and reused, i. e. those in the Krebs Cycle. Some damages such as DNA mutations are caused by enzymatic mistakes and are the source of evolution, and these mistakes might well be evolved selectively (not randomly) to increase the fitness of species. Please list a few damages showing the diversity of damages. Please explain, using DNA damage as an example, how the age-related changes can be both random and programmed.

Figure 1: The bottom right box does not show the same subject as the box on the bottom left.
Figure 2: Would you integrate the Gibbs free energy theory in this Figure to explain the energy-entropy relation during the aging process? [formatting query]
Table 1. Please add a table, as suggested by the reviewer, including the AP genes of various types, and the damages generated by their functions in cells, tissue, and organisms of later life. A few have been listed in the text such as mTOR, growth hormone receptor, and telomerase.
Table 2. Please list some cases of gene names about seesaw model, as suggested by the reviewer.

1st Review	12/04/2020

Reviewer(s)' Comments to Author:

Reviewer: 1

Comments to the Author

This is a very nice and timely review in ageing area and it will help us to understand ageing from a new perspective. At first, the authors talk about current understanding on ageing and longevity. Then the authors from genetics and evolution perspective to discuss damage is a main driving factor for ageing. Furthermore, the authors proposed seesaw model to divide ageing into “pro-stemness” and “pro-function” two states based on the damage production and dilution. Finally, the authors discuss the current approaches to rejuvenation including reprogramming and regeneration.
Although this review discussion is both deeply and comprehensive, I still have some concerns listed below:

1) Page3-6, the first parts about ageing and longevity; In fact, I always want to know the differences between ageing and longevity if they are two different phenotypes. Therefore, if the authors would like to spend a little parts talking about the relationship between ageing and longevity, it will make this review better than before;
2) Page10 about AP genes; I’m very interested in this part and really enjoy reading it. I would like to suggest the authors to make a table to list some classical or key AP genes which I believe will attract lots of readers.
3) Page 11 about seesaw model. This model is very interesting and reasonable. Is it possible to list some cases like gene names on the model(Figure4);
4) Page 15-17 about rejuvenation through reprogramming. It looks like that only one case is effective in using Yamanaka factors to prevent ageing. So if the authors know other similar cases, it may be better to talk a little bit about them. Another suggestion is spending a little part talking about the relationship between niche and reprogramming and it will make reprogramming parts more attractive.

Reviewer: 2

Comments to the Author
In this manuscript, Zhang and Gladyshev have reviewed large quantity of studies and proposed their theories of rejuvenation. They discussed several mechanisms of aging with focusing on rejuvenation by reprogramming. Overall, the review is interesting and reads well, which provides solid information and ideas in the field. However, some arguments are one sided and counter-points discussion in a few areas would enrich the manuscript.

1. Methods to investigate aging and rejuvenation are important in this field. The authors discuss a few published methods of rejuvenation such as parabiosis and HSC transplant. These areas are controversial. It is suggested that limitations of these methods should also be provided to the readers. For example, while studies have provided evidence that parabiosis leads to animal rejuvenation, the mechanisms are not simple. As discussed by Conboy and colleagues, a single blood exchange between young and old mice provides minor benefits to old mice while young mice exhibit impaired healing responses. This raises a question of whether young mice carry pro-rejuvenation factors or the aged blood carries inhibitory compounds. For BMT studies assessing HSC transplant the methods used to transplant the cell are often extreme, such as irradiation which itself can initiate DNA damage. Therefore while the donor cells follow the donor cell age, the recipient age is likely worse off than before transplant. Therefore while these models have been used in the past there are a number of factors that must be consider before branding these as rejuvenation therapies, as this leads to over simplification of these complex topics.

2. The idea that aging is reset to zero for generation assumes that the somatic cells exhibit the same aging characteristics as the germline cells. Are there studies comparing the aging of these two systems and looking at disparities between the two? The idea is suggested that increased cell growth would help dilute the damage, however recent studies suggest that increased cell growth can trigger senescence by cytoplasmic dilution. How is cell growth distinct from cell proliferation in diluting damage?

3. To follow up on the idea that proliferation may help dilute damage, how do the authors believe that this relates to bone marrow stem cells, which are present in a niche rich in stem cell pro-stemness factors. In this scenario the function of the cells is to maintain stemness in order to produce all blood lineage cells, however sacrificing stemness for biological function would be a loss of the primary cell function. Do the authors believe that this idea is applied to all adult stem cell niches or that it occurs in an organ specific context?

4. During the induction of iPS cells to a biological age of zero the authors mention that this gradually reverses age-related damage. Mutations are one form of age related damage. Is there evidence of iPS cells correcting mutations upon reprogramming? Age-related damage should be more specifically defined to provide better context of the evidence in the literature.

Reviewer: 3

Comments to the Author
In this perspective paper, “How Can Aging be Reversed? Exploring Rejuvenation from a Damage-Based Perspective” the authors present a new model for understanding how cells age and why iPS cells and regeneration are both valid strategies for

rejuvenation. A key proposal in the manuscript is the “seesaw model” describing cells as having two potential states, pro-stemness and pro-function, and how cells move between the two states, accumulate damage, and how damage is reset in different contexts. The model also captures the issues with regards to pushing the seesaw too far in either direction leading to issues such as cancer or senescence. The authors often find nice references to support their presented hypothesis, but do not present potential counter-arguments or the literature supporting alternative interpretations. A more robust development of the authors’ main model presented would provide a welcome model that would be highly relevant to aging researchers.
Major Points:
1) As presented, the title and abstract are not accurately reflected in the current manuscript. A revised title and abstract could better capture the reviewers’ interpreted focus of this perspective-- which was the presentation of the “seesaw model” to model and how iPS cells and regeneration strategies allow cells to be rejuvenated. Because the seesaw model was seen as a major focus of this perspective, Figure 4 stood out as a key figure. However, the current figure layout is difficult for the reader to follow. It would be clearer to the reader if the figure was made more concise.
2) It is unclear what types of cells are being discussed throughout the manuscript- the proposed model of pro-function vs pro-stemness in “cells” appears to be a general model- but often in examples seems to be describing functions / actions of adult stem cells- and thus this model is perhaps a version of differentiation vs self-renewal in stem cells. This is never explicitly stated and needs to be clarified to better interpret the presented model. Also, the lines are a bit blurry between development and aging (perhaps intentionally) but could be made more clear when discussing “early life” especially in the wound healing section.
3) There seem to be a few instances (see below for a few) in which the references seem to be out of place (or critically missing)
A) In the argument for shifting the system towards “pro-function” by the overexpression of p53- the reference uses a mutant form of p53 and those authors wrote a correction in 2005 stating there were several other factors that could have contributed to the shortened life-span.
B) Also, in the section on interventions restoring function to aged cells, they cite a manuscript showing essentially the opposite- where cell intrinsic properties of aged stem cells do not get reset in young environments (pg 11 Soraas, A et al reference) Perhaps including a parabiosis reference could be relevant instead.
C) Please include references for hematopoietic stem cell exhaustion and aging acceleration
4) The figures in general do not necessarily contribute to the understanding of the text. For instance, figure 2 could be used to visually describe the section on aging and entropy- which would be helpful. However, it is not clear how this figure helps explain the restricted exchange tied to age and size (again reference here would be useful) that was proposed.
5) In the section addressing DNA damage dilution / removal the authors make the argument of damage dilution through proliferation; however, there is a substantial body of literature suggesting that the proliferation/cell division process generates significant amounts of damage. Please minimally address this contradiction.
6) One section in particular lead to some confusion- on pg 5 of the manuscript.
A) In the discussion of trade-offs of increased lifespan, caloric restriction should be discussed and referenced. The authors have chosen to use reproductive capacity as “the” measure of fitness, but only discuss this metric once near the end of the paragraph --while providing many other metrics of fitness. We suggest the authors remove “most notably reproductive capacity“.
B) With regards to the statement that there is no selective pressure for genes that promote longevity, minimally the opposing viewpoint should be addressed and referenced. Perhaps cite/ address the following papers: H. Chen, A. Maklakov 2012, S. Williams, M. Shattuck 2015, and A. Maklakov et al. 2015.
C) In the final sentence of page 5, “This” needs to be clarified, as the previous sentence discusses trade-offs. Please clarify what is associated with “the decline in the force of selection with age.”
7) The logical progression in several paragraphs in the manuscript tend to be roundabout to reach the main idea. Reorganization of many sections would lead to a more linear trajectory towards the paragraphs’ thesis and make the prospective easier to follow.

Minor Points:
There were grammar and spelling errors throughout the manuscript which should be revised.

On page 3 of the manuscript, the authors state that “aging is a superposition of systematic and random changes.” However, a superposition implies that the systemic and random changes associated with aging are simply a sum of the two changes. Therefore, the evidence for a superposition should be referenced or perhaps different word choice could be used to suggest a combination of the two types of changes.

References chosen for the interventions that increase lifespan of model organisms (pg 5) should encompass a broader range of available interventions. The references should include caloric restriction, senolytics, Sirtuins, genetic models, and partial reprogramming. Also given the large body of literature on each, perhaps it would be sufficient to cite current reviews on each intervention.

It is unclear what the authors mean by young blood transfusions bringing dilution of damage and "pro-stemness" in the comparison to stem cell proliferation (pg 16) - it reads a bit like young blood transfusions are adding younger stem cells but this may be simply the clarification of which cells are being more "pro-stem"

On page 7, the first paragraph should cite Schrodinger's definition of life.

On page 8, please clarify whether the entropy statements are with regards to inter or intra species comparisons.

The discussion of genetic damage (bottom of pg 9) could be expanded upon. It seems that the authors are stating that once a mutation is in the genome- the damage it generates is no longer random damage- as it's programmed now in the DNA. However, is the initial damage random?

On page 10, all genes are described as AP-like, however, mutations of certain genes lead to increased fitness or longevity exist which the authors recognize as anti-AP genes. The above statement should be corrected, since not all genes are AP-like.

The paragraph on page 12 is very important to the focus of the perspective. However, much of the background information was not established earlier in the manuscript. While the authors put a great deal of effort into laying the foundation for explaining their seesaw model, we suggest that more focus is given to the background required to fully grasp the contents of this paragraph and to expand upon the contents of this paragraph.

Section on wound healing, there is an omission of how scaffolds and biomaterials can improve healing vs scar tissue- and again only a presentation and support of the authors hypothesis.

Author response to 1st review and editorial recommendations	01/27/2020

We would like to thank reviewers for constructive feedback and suggestions, which helped us to improve the manuscript. Please see our point-by-point responses below.

Reviewer 1:

Comment: This is a very nice and timely review in ageing area and it will help us to understand ageing from a new perspective. At first, the authors talk about current understanding on ageing and longevity. Then the authors from genetics and evolution perspective to discuss damage is a main driving factor for ageing. Furthermore, the authors proposed seesaw model to divide ageing into "pro-stemness" and "pro-function" two states based on the damage production and dilution. Finally, the authors discuss the current approaches to rejuvenation including reprogramming and regeneration. Although this review discussion is both deeply and comprehensive, I still have some concerns listed below:
Response: We would like to thank the reviewer for his/her interest in our work, thoughtful recommendations, and detailed feedback. We modified the text to more clearly describe our ideas.

Comment: 1) Page 3-6, the first parts about ageing and longevity; In fact, I always want to know the differences between ageing and longevity if they are two different phenotypes. Therefore, if the authors would like to spend a little parts talking about the relationship between ageing and longevity, it will make this review better than before;
Response: Thank you. We added text on pages 3-4 on difference between aging and longevity. We also comment that a shortened lifespan is associated, with but does not necessarily mean accelerated aging.

Comment: 2) Page10 about AP genes; I'm very interested in this part and really enjoy reading it. I would like to suggest the authors to make a table to list some classical or key AP genes which I believe will attract lots of readers.
Response: Thank you. We think that all genes have antagonistic pleiotropy properties. We have made a new table (Table 2) describing the functional and deleterious effects of some genes and processes and discuss them in more detail.

Comment: 3) Page 11 about seesaw model. This model is very interesting and reasonable. Is it possible to list some cases like gene names on the model (Figure4);
Response: We include Table 2 with such examples.

Comment: 4) Page 15-17 about rejuvenation through reprogramming. It looks like that only one case is effective in using Yamanaka factors to prevent ageing. So if the authors know other similar cases, it may be better to talk a little bit about them.

Another suggestion is spending a little part talking about the relationship between niche and reprogramming and it will make reprogramming parts more attractive.
Response: Thank you. We added similar cases including SCNT and generation passage through reproduction. Also, a recent work that applies the Horvath clock (page 6) suggests a possibility of achieving rejuvenation. We extended text on this topic. The question about the niche is particularly interesting and we also added text in this section.

Reviewer 2:

Comment: In this manuscript, Zhang and Gladyshev have reviewed large quantity of studies and proposed their theories of rejuvenation. They discussed several mechanisms of aging with focusing on rejuvenation by reprogramming. Overall, the review is interesting and reads well, which provides solid information and ideas in the field. However, some arguments are one sided and counter-points discussion in a few areas would enrich the manuscript.
Response: We would like to thank the reviewer for these comments.

Comment: 1. Methods to investigate aging and rejuvenation are important in this field. The authors discuss a few published methods of rejuvenation such as parabiosis and HSC transplant. These areas are controversial. It is suggested that limitations of these methods should also be provided to the readers. For example, while studies have provided evidence that parabiosis leads to animal rejuvenation, the mechanisms are not simple. As discussed by Conboy and colleagues, a single blood exchange between young and old mice provides minor benefits to old mice while young mice exhibit impaired healing responses. This raises a question of whether young mice carry pro-rejuvenation factors or the aged blood carries inhibitory compounds. For BMT studies assessing HSC transplant the methods used to transplant the cell are often extreme, such as irradiation which itself can initiate DNA damage. Therefore while the donor cells follow the donor cell age, the recipient age is likely worse off than before transplant. Therefore while these models have been used in the past there are a number of factors that must be consider before branding these as rejuvenation therapies, as this leads to over simplification of these complex topics.
Response: Thank you for these insights on possible rejuvenation therapies. We agree that both parabiosis and HSC transplantation are just a direction towards a potential rejuvenation rather than a well-established therapy. We toned down the discussion in this section. Also, we are aware that the recipient lifespan may not be extended in terms of bone marrow transplantation and added some text on this issue.

Comment: 2. The idea that aging is reset to zero for generation assumes that the somatic cells exhibit the same aging characteristics as the germline cells. Are there studies comparing the aging of these two systems and looking at disparities between the two? The idea is suggested that increased cell growth would help dilute the damage, however recent studies suggest that increased cell growth can trigger senescence by cytoplasmic dilution. How is cell growth distinct from cell proliferation in diluting damage?
Response: Thank you. There are studies comparing somatic cells and germline cells, or differentiated cells and ES cells. A key problem is that it is difficult to separate aging characteristics from development-related features. So far, the only reliable way to follow progression through aging is by using aging biomarkers. We are currently working on aging characteristics of germline cells by using DNA methylation clocks. The question about damage dilution and damage generation through proliferation is an important and thought-provoking point. We think that damage dilution may be related to increased cell proliferation but not the other way round. Whether cell proliferation is related to damage dilution may depend on cell state.

Comment: 3. To follow up on the idea that proliferation may help dilute damage, how do the authors believe that this relates to bone marrow stem cells, which are present in a niche rich in stem cell pro-stemness factors. In this scenario the function of the cells is to maintain stemness in order to produce all blood lineage cells, however sacrificing stemness for biological function would be a loss of the primary cell function. Do the authors believe that this idea is applied to all adult stem cell niches or that it occurs in an organ specific context?
Response: Thank you. For the bone marrow, we cite one relevant paper, which found that when cells are forced to proliferate, the lifespan is shortened. This is in an organ-specific manner - we do not have evidence that it happen to all stem-cell niches as adult stem cells are usually fueled by different factors. We added some text on this issue.

Comment: 4. During the induction of iPS cells to a biological age of zero the authors mention that this gradually reverses age-related damage. Mutations are one form of age related damage. Is there evidence of iPS cells correcting mutations upon reprogramming? Age-related damage should be more specifically defined to provide better context of the evidence in the literature.
Response: Thank you. The reviewer is right in that mutations are irreparable and this should have been mentioned in the review. In this sense, not all damage is dilutable. However, mutations may be diluted on the evolutionary timescale by the process of natural selection. We have made this point in the revised manuscript.

Reviewer 3:

Comment: In this perspective paper, "How Can Aging be Reversed? Exploring Rejuvenation from a Damage-Based Perspective" the authors present a new model for understanding how cells age and why iPS cells and regeneration are both valid strategies for rejuvenation. A key proposal in the manuscript is the "seesaw model" describing cells as having two potential states, pro-stemness and pro-function, and how cells move between the two states, accumulate damage, and how damage is reset in different contexts. The model also captures the issues with regards to pushing the seesaw too far in either direction leading to issues such as cancer or senescence. The authors often find nice references to support their presented hypothesis, but do not present potential counter-arguments or the literature supporting alternative interpretations. A more robust development of the authors' main model presented would provide a welcome model that would be highly relevant to aging researchers. Major Points:

Response: We would like to thank the reviewer for suggestions which support discussion of these topics and help us to improve the manuscript.

Comment: 1) As presented, the title and abstract are not accurately reflected in the current manuscript. A revised title and abstract could better capture the reviewers' interpreted focus of this perspective-- which was the presentation of the "seesaw model" to model and how iPS cells and regeneration strategies allow cells to be rejuvenated. Because the seesaw model was seen as a major focus of this perspective, Figure 4 stood out as a key figure. However, the current figure layout is difficult for the reader to follow. It would be clearer to the reader if the figure was made more concise.

Response: Thank you. We modified the abstract as well as the figure. We opted to keep the original title.

Comment: 2) It is unclear what types of cells are being discussed throughout the manuscript- the proposed model of pro-function vs pro-stemness in "cells" appears to be a general model- but often in examples seems to be describing functions / actions of adult stem cells- and thus this model is perhaps a version of differentiation vs self-renewal in stem cells. This is never explicitly stated and needs to be clarified to better interpret the presented model. Also, the lines are a bit blurry between development and aging (perhaps intentionally) but could be made more clear when discussing "early life" especially in the wound healing section.

Response: Thank you. We think that the "functions" here is a broader feature than simply self renewal and differentiation. We tried to describe it through examples instead of giving a clear definition of which feature is "pro-function" or "pro-stemness". We added some text discussing early life aging and development, as this question is particularly interesting to us.

Comment: 3) There seem to be a few instances (see below for a few) in which the references seem to be out of place (or critically missing)

A) In the argument for shifting the system towards "pro-function" by the overexpression of p53- the reference uses a mutant form of p53 and those authors wrote a correction in 2005 stating there were several other factors that could have contributed to the shortened life-span.

Response: Thank you. Multiple models of super p53 overexpression have been developed in addition to the Tyner et al. paper. Considering that tumor incidence (key cause of mortality in lab mice) is lowered in these models, they do display a faster aging speed. We added discussion to further clarify this point.

Comment: B) Also, in the section on interventions restoring function to aged cells, they cite a manuscript showing essentially the opposite- where cell intrinsic properties of aged stem cells do not get reset in young environments (pg 11 Soraas, A et al reference) Perhaps including a parabiosis reference could be relevant instead.

Response: Thank you for pointing thus out and sorry for the confusion. This citation is to show that the age of the reconstituted blood follows the age of the donor. The same mechanism may work for the young donor as well. We added text to avoid misunderstanding.

Comment: C) Please include references for hematopoietic stem cell exhaustion and aging acceleration.

Response: Thank you. We modified the text and included Kirschner et al. 2017 paper. We also included additional citations on the topic (Rossi et al. 2007).

Comment: 4) The figures in general do not necessarily contribute to the understanding of the text. For instance, figure 2 could be used to visually describe the section on aging and entropy- which would be helpful. However, it is not clear how this figure helps explain the restricted exchange tied to age and size (again reference here would be useful) that was proposed.

Response: Thank you. We modified the figure.

Comment: 5) In the section addressing DNA damage dilution / removal the authors make the argument of damage dilution through proliferation; however, there is a substantial body of literature suggesting that the proliferation/cell division process generates significant amounts of damage. Please minimally address this contradiction.
Response: Thank you. We think that cell proliferation is a pro-stemness process. However, whether cell proliferation is related to the damage dilution will depend on the cell state. In some conditions, e.g. fibroblast proliferation in cell culture, damage accumulates, in others it is diluted. We addressed this issue further in the text.

Comment: 6) One section in particular lead to some confusion- on pg 5 of the manuscript.
A) In the discussion of trade-offs of increased lifespan, caloric restriction should be discussed and referenced. The authors have chosen to use reproductive capacity as “the” measure of fitness, but only discuss this metric once near the end of the paragraph --while providing many other metrics of fitness. We suggest the authors remove “most notably reproductive capacity“.
Response: Thank you. We added referenced and removed the sentence.

Comment: B) With regards to the statement that there is no selective pressure for genes that promote longevity, minimally the opposing viewpoint should be addressed and referenced. Perhaps cite/ address the following papers: H. Chen, A. Maklakov 2012, S. Williams, M. Shattuck 2015, and A. Maklakov et al. 2015.
Response: Thank you for this point. We cited these papers.

Comment: C) In the final sentence of page 5, “This” needs to be clarified, as the previous sentence discusses trade-offs. Please clarify what is associated with “the decline in the force of selection with age.”
Response: Thank you. We modified the text to clarify “this” as a relationship between longevity and fitness.

Comment: 7) The logical progression in several paragraphs in the manuscript tend to be roundabout to reach the main idea. Reorganization of many sections would lead to a more linear trajectory towards the paragraphs’ thesis and make the prospective easier to follow.
Response: Thank you. We hope the changes we’ve made throughout the text make the text easier to read.

Comment: Minor Points:
There were grammar and spelling errors throughout the manuscript which should be revised.
On page 3 of the manuscript, the authors state that “aging is a superposition of systematic and random changes.” However, a superposition implies that the systemic and random changes associated with aging are simply a sum of the two changes. Therefore, the evidence for a superposition should be referenced or perhaps different word choice could be used to suggest a combination of the two types of changes.
Response: Thank you. We modified the text.

Comment: References chosen for the interventions that increase lifespan of model organisms (pg 5) should encompass a broader range of available interventions. The references should include caloric restriction, senolytics, Sirtuins, genetic models, and partial reprogramming. Also given the large body of literature on each, perhaps it would be sufficient to cite current reviews on each intervention.
Response: Thank you. We updated citations to include these interventions.

Comment: It is unclear what the authors mean by young blood transfusions bringing dilution of damage and “pro-stemness” in the comparison to stem cell proliferation (pg 16) - it reads a bit like young blood transfusions are adding younger stem cells but this may be simply the clarification of which cells are being more "pro-stem"
Response: We think that young blood brings the environment that supports stem cells.

Comment: On page 7, the first paragraph should cite Schrodinger’s definition of life.
Response: Thank you. We updated the citation to include it.

Comment: On page 8, please clarify whether the entropy statements are with regards to inter or intra species comparisons.
Response: Thank you. We wrote it in an intra-species context but it should apply to all species that age.

Comment: The discussion of genetic damage (bottom of pg 9) could be expanded upon. It seems that the authors are stating that once a mutation is in the genome- the damage it generates is no longer random damage- as it’s programmed now in the DNA. However, is the initial damage random?

Response: We think the initial damage is random, with certain sites being more susceptible to damage. We expanded the discussion genetic damage in several places.

Comment: On page 10, all genes are described as AP-like, however, mutations of certain genes lead to increased fitness or longevity exist which the authors recognize as anti-AP genes. The above statement should be corrected, since not all genes are AP-like.
Response: Thank you. We think that while all genes have AP-like properties (i.e. they are beneficial, but their use contributes to cumulative damage, some genes may appear as anti-AP genes when considered against the overall damage trajectory.

Comment: The paragraph on page 12 is very important to the focus of the perspective. However, much of the background information was not established earlier in the manuscript. While the authors put a great deal of effort into laying the foundation for explaining their seesaw model, we suggest that more focus is given to the background required to fully grasp the contents of this paragraph and to expand upon the contents of this paragraph.
Response: Thank you. We added some transitions to make it easier to read.

Comment: Section on wound healing, there is an omission of how scaffolds and biomaterials can improve healing vs scar tissue- and again only a presentation and support of the authors hypothesis.
Response: Thank you. We updated the discussion about this.

[Author replies to editorial recommendation]
Editorial Recommendation:

This is a thought-provoking review that discusses several fundamental questions in the aging biology, proposes a nice damage-based seesaw model, and suggests new directions to reverse the aging process. The authors point out the different mechanisms between de-differentiation and rejuvenation, and that different reversal strategies are needed to the cells at "pro-stem" and "pro-function" state. They introduce a unified aging model, attempting to explain the aging process at cellular, tissue, and organism levels. However, the model could be more carefully specified by adding examples of genes that promote stemness or (commitment, differentiation?) "function". Some of the roles of damage and niche signals in resetting age in stem cells and (their model) iPSC could be discussed. Reviewers are thoughtfully engaged and provide excellent and constructive comments. All referee comments are useful and should be addressed. As to that there was not enough critical thinking about the opposing views, we would not insist upon balance unless the piece is to be longer Review (which would probably increase its impact). The Perspective can well be one-sided, but not unskeptical. Please consider our comments on the specific referee points and additional recommendations:
Response: We would like to thank the editor for recruiting highly qualified and constructive reviewers as well as for the extensive editorial suggestions and comments. We much appreciate the effort to help us to make the most impactful paper. We changed the name of the seesaw model to stemness-function model to better reflect its key features in its name. We also modified the text significantly in response to both reviewers' and editor's suggestions.

(1) Please address the differences between aging and longevity
Response: Thank you. We discuss now that aging is a process of damage accumulation, whereas longevity is related to the question of how long organisms live. We make clear differences between them. We also discuss that interventions that slow down the rate of aging do not necessarily affect lifespan, and those that affect lifespan do not necessarily change the rate the aging, as they may target a particular lethal disease. Therefore, although lifespan studies still represent a convenient way to assess the effects on aging, development of biomarkers that directly quantify the aging process is also necessary.

(2) The genetic programing of aging: Regarding if aging is a genetically programmed process, the authors concluded that it is "a combination of predictable transitions (program-like) and random events". The question is whether the different aging rates among tissues have distinct roles in the lifespan.
Response: This is a really good question. Some cells and tissues may age slower. This issue has not yet been thoroughly addressed experimentally, because there have been no sufficiently accurate and universal biomarkers to assess the difference in the aging rates between different tissues. As discussed in the manuscript, various clocks have recently been developed, and we hope they will be used to address this question in the future. In our previous work, we found that human reproductive tissues age faster than other tissues (Podolskiy et al 2016).

(3) Adding a table of the AP genes can help explain the tradeoff between the fitness in young lives and longevity or aging. This will make the paper more useful and increase its impact. Some of the best examples are in model organisms. Those that benefit the fitness seem to be driven by reproductive success. You might also want to explain the "deleterious" phenotypes caused by these genes in old organisms.
Response: Thank you. We added a table that lists AP properties of several genes and processes.

(4) Stress vs. lifespan: To be convincing, you might want to provide examples of harmful phenotypes imposed by mild stress that extend the lifespan. This will also help stress your point that this does not fit the old AP theory. High stress may increase the biological age and shorten the lifespan (doi: 10.1016/j.arr.2018.10.001), which fits the AP theory. It is possible that mild stress activates different sets of genes from high stress, and both have opposite effects on lifespan. For example, in plants, high, ambient, and cold temperatures can activate different sets of miRNAs and their targeting genes.
Response: Thank you for the suggestion. This article provides a good point regarding mild and extreme stresses. This is also seen in previous studies in the case of 20% and 40% CR (doi: 10.1016/j.cmet.2016.05.027), wherein extreme CR cancels the longevity effect. We cited the article and added discussion.

(5) List some cases of genes on the seesaw model to explain dilution of damage. According to the seesaw theory, the damage can be diluted during frequent proliferation in embryo and germ cells. What are the experimental supports for this? How does the dilution explain that the DNA repairing plays a pivotal role in maintaining low mutation rate in germ and stem cells (doi: 10.1038/ncomms15183)? Are good cells selectively propagated over damaged cells due to cell cycle arrest? This raises interesting questions about whether and how damaged genomes provide a proliferative stimulus to stem cells and this could be a fascinating extension to the review – this needs to be explained using experiments. In addition, a study showed aging cells can simply dump the cytoplasm containing the damage components (doi: 10.1038/nature21362) - This might well be an alternative way of dilution.
Response: Thank you. We added the relevant discussion including a few cases. Regarding the experimental support, it has been shown that Shannon entropy and methylation age are both decreased until a certain point during embryonic development. Regarding low mutation rate in germ and stem cells, we think the difference between mutation rate in somatic and germ stem cells is due to the DNA damage response activity and mutation rate in cell proliferation, both relevant to the original damage. The damage rate between iPSCs and somatic cells are not known yet. We are currently collaborating with Jan Vijg lab to figure this out. Dumping the damage is indeed an additional mechanism to dilute the damage. However, simply dumping the damage to the extracellular milieu may cause additional damage in the cell niche in more complex organisms, possibly resulting in inflammation. Therefore, it may be a source of accelerated aging ("inflammaging") rather than what it is designed to be as a form if dilution alternatives.

Does the damage dilution help erase and reestablish DNA methylation during the embryonic reprogramming? In cancer cells, DNA damage and epigenomic landscape intertwines to promote cellular growth and proliferation (Please briefly comment on this, as this can be a major review).
Response: Thank you. We basically treat DNA methylation status as a feature that marks the level of damage. This decline is observed after proliferation (see Olova et al. 2019), but without further evidence we can only claim correlation but not causation. The key problem is that this hypothesis cannot be tested in adult fibroblasts, as they accumulate more damage than they dilute and show increased methylation age. We are not sure how damage dilution helps reinstate the youthful epigenome. It may be that the cells state governs both damage dilution and epigenome reprogramming. In cancer cells, aging becomes tricky: cancer cells have distinct methylation ages compared to normal cell types, with either significantly higher or lower methylation age.

It might also be useful to discuss the case of Dolly, the sheep, who have the DNA mutations (damage) in the pro-function state nucleus of mammary gland cell been diluted after being transferred to an unfertilized oocyte (nucleus was removed) and proliferating during embryonic development in the apparently pre-stemness environment.
Response: Thank you. We added some discussions on SCNT. However, the case of Dolly the sheep remains controversial as it displayed age-related phenotypes while other cloned organisms do not necessarily have this issue. We planned to discuss some possibilities that happens to Dolly such as occasional key mutations happen that causes aging acceleration (for example, DNA damage repair genes) but the technique at that point is not adequate to address this question.

(6) Re-setting the aging clock: Discuss briefly the embryonic reprogramming as re-setting the aging process and discuss the role of stem cell niches in providing the equivalent of reprogramming signals.
Response: Thank you. Basically the reprogramming factors have been chosen. Admittedly, there are many similar stem cell niches that recapitulate this effect. Usually, these are fueled by the pathways promoting stemness of stem cells (e.g. Wnt). There has been an article discussing the DNA methylation profiles in an organoid system mimicking stem cell niches, and from our preliminary studies many adult stem cells have different methylation profiles compared to tissue samples. We think it would be interesting to apply the clock to organoids to see if the stem cell niche could affect the methylation age.

(7) Parabiosis and classical "rejuvenation therapies" are complex to interpret and should not just be labeled "regeneration". In terms of iPS reprogramming resets aging. Should there therefore be analysis or at least comment on the role of c-Myc?

Response: Thank you for the discussion on c-Myc. New studies from the Sinclair lab in collaboration with our lab (Lu et al. 2019) have shown that only OSK expression may be able to reverse methylation age without c-Myc. But full details remain unclear.

(8) The analogy between aging and entropy: You might want to define several thermodynamics-related concepts to help the reader follow the discussion, for example, the law of thermodynamics, “increased variability during aging”, “methylation entropy”, “isolate system” vs. “open system”. Please cite “What is life?” explaining why Schrödinger did not think that life followed the physical laws.
Response: Thank you. We made a few clarifications to the text and added the citation. In the Schrödinger’s book, it is discussed that animals obtain the “order” from foods, which have ordered structures. Our explanation is similar. In addition, we realized that the less “ordered” food (for example, food from older animals) will give the animals less “order”. We tried to address this by feeding organisms food based on old and young organisms.

Please explain how the negative entropy flow happens during the aging process. On the top of the suggestion that the negative entropy flow was caused by the inhibition of energy exchange and distribution, could you also comment on how the reduction of NAD and energy decline (enthalpy, ΔH, reduced?) in aging cells contributes to the increase of entropy? Entropy is a measure of energy dispersal after all. According to $\Delta G = \Delta H - T\,\Delta G$, if Gibbs free energy, ΔG, is negative, then the reaction is spontaneous with increased entropy. The decline of energy exchange could result from reduced energy production. For example, DNA damage or somatic mutation accumulation can increase the genomic entropy, and this might be due to the energy decline, because repairing, removal of dying cells, and energy exchanges and distribution are all energy-driven processes. Heterochromatin loss and hypermethylation and activation of transposons, and increased DNA damages during the aging process and cellular enlargement are good examples to explain the increase of entropy during aging.
Response: Thank you for this in-depth discussion. We have integrated some of it into our manuscript. This part may work better in the section discussing the damage than discussing entropy. We think that living systems maintain entropy by material exchange with the outside world. A living system has to take in substances with low entropy to maintain low entropy flow. It is beneficial to mention the spontaneousness of reactions, we think that inability to take in substances from outside environment and the disrupted mitochondrial functions limit the ability to process some endothermic biochemical reactions, and the existing damage limits the ability to complete some slow reactions. Altogether, this limits the ability to repair damage. In terms of NAD+ decline, we are not sure how it contributes.

Schroedinger is relevant, but extended discussion of Shannon’s idea of information and entropy on opposite sides of a possibly different seesaw would need some discussion of how Shannon’s information and entropy can be measured in aging cells, for example (Slieker 2016 10.1186/s13059-016-1053-6, Jenkinson 2017 10.1038/ng.3811). In this paper, https://doi.org/10.1186/s13059-019-1753-9, the authors showed the Horvath clock CpG methylation sites are of high entropy, or high variability, perhaps suggesting that some biological processes are specifically vulnerable for the changes during the aging process.
Response: Thank you for mentioning this useful paper. We read it added some discussion on Shannon entropy. Previously, we published a research paper that showed the increase of Shannon entropy during aging (doi: 10.1111/acel.12738.). Interestingly, the entropy effects differed for the sites that increased, decreased and did not change methylation with age. For the high entropy on Horvath CpG sites, we think that this may be explained by the trend for the methylation to go to 0.5 with age.

(9) Damages are caused by the side products of the reactions. In organisms, enzymatic reaction side products are often recycled and reused, i. e. those in the Krebs Cycle. Some damages such as DNA mutations are caused by enzymatic mistakes and are the source of evolution, and these mistakes might well be evolved selectively (not randomly) to increase the fitness of species. Please list a few damages showing the diversity of damages. Please explain, using DNA damage as an example, how the age-related changes can be both random and programmed.
Response: Thank you. We mainly talked about the by-product generated through abnormal functions. There are for sure a great number of by-products generated by normal biological processes, and therefore the biological system has ways to cope with them. We cited an article (doi: 10.1016/j.tibs.2019.07.004) and explained by using glycolysis as an example. Basically, all pathways mentioned have by-products, but not all of them are efficiently reused.

Figure 1: The bottom right box does not show the same subject as the box on the bottom left.
Response: Thank you. We modified.

Figure 2: Would you integrate the Gibbs free energy theory in this Figure to explain the energy-entropy relation during the aging process? [formatting query]
Response: Thank you. We modified the text.

Table 1. Please add a table, as suggested by the reviewer, including the AP genes of various types, and the damages generated by their functions in cells, tissue, and organisms of later life. A few have been listed in the text such as mTOR, growth hormone receptor, and telomerase.
Response: Thank you. We added this table.

Table 2. Please list some cases of gene names about seesaw model, as suggested by the reviewer.
Response: Thank you. We listed some manipulations about the model as described in the manuscript.

2nd Editorial decision	02/12/2020

Editor Comments to Author:

Thank you for spending time to revise the manuscript. As you indicate, the aging field needs an organized theory. We appreciate very much for your efforts for suggesting a new model and that your piece sheds a new light in the field by raising questions and proposing new directions. There are some discussions, however, that still need to be clarified, based on our assessment. In the attached manuscript, please find our edits, questions, and suggested changes. Here is a summary of our major points:

(1) It was not clear to us if the damage dilution refers to the DNA damage or some other damages or both. The statements about the damages are rather vague. It would be helpful if you can list the concrete damages (if not the DNA damage) and how they are diluted, ideally with supporting references or your own observations. We found that even for the byproducts from biochemical reactions in the cells, which might impose damages, there were no supporting references showing their dilutions during embryonic development or stem cell proliferations. In your revised manuscript, please add this information.

Since we are a genetic journal, we would like to see the discussion more focused on the damages of the genome or DNA or RNA to fit the journal's scope.

(2) It is well-known that the DNA damage is accompanied by the aging process. Several recent papers have shown that during the embryonic development, the DNA damage sensing and DNA repairing systems are rather relaxed (Kermi et al 2019, doi:10.3390/genes10050398), which allows these early cells to proliferate frequently and fast, as a consequence, DNA damages accumulate. This does not seem consistent with the damage dilution model that you proposed. Perhaps you did not mean the DNA damage, but other damages? Please clear this up.

(3) Some references suggest that the cell proliferation of germ cells is accompanied by apoptosis in a large portion of these germ cells, and their exiting from cell cycle into the differentiation of oocytes preludes with cell cycle arrest allowing the DNA damage sensing and repairing - This has been an established mechanism that "dilute" or remove the DNA damage from gametes. [suggested references]
Additional mechanism has also been shown very recently to reduce mutations in germ cells through transcriptional scanning: Xia et al doi: 10.1016/j.cell.2019.12.015

Thus, you might want to modify the damage dilution model by adding these mechanisms and references to clarify your point. Apparently, the DNA damage is diluted through these mechanisms.

(4) Embryonic stem cells and other stem cells contain inner immunity function, i. e. these cells can function like non-professional phagocytes to remove their neighboring apoptotic cells and cell debris without eliciting inflammatory response. This has been shown repeatedly in worms, flies, and mammals under EM.

(5) You might want to add some examples of the AP genes in four categories that you discussed in the text.

(6) [discussion with author on possible titles]

2nd Author response to editorial recommendations	03/02/2020

[Editor recommendation]
Thank you for spending time to revise the manuscript. As you indicate, the aging field needs an organized theory. We appreciate very much for your efforts for suggesting a new model and that your piece sheds a new light in the field by raising questions and proposing new directions. There are some discussions, however, that still need to be clarified, based on our

assessment. In the attached manuscript, please find our edits, questions, and suggested changes. Here is a summary of our major points:

Response: We would like to offer our greatest appreciation to the editors for helping us to improve the article and bringing out fascinating new perspectives. Not only these helped us to consider the anti-aging approaches from a different angle, they also allowed us helped to in designing future studies. We carefully considered the editors' suggestions and modified the manuscript as discussed follows.

(1) It was not clear to us if the damage dilution refers to the DNA damage or some other damages or both. The statements about the damages are rather vague. It would be helpful if you can list the concrete damages (if not the DNA damage) and how they are diluted, ideally with supporting references or your own observations. We found that even for the byproducts from biochemical reactions in the cells, which might impose damages, there were no supporting references showing their dilutions during embryonic development or stem cell proliferations. In your revised manuscript, please add this information.
Since we are a genetic journal, we would like to see the discussion more focused on the damages of the genome or DNA or RNA to fit the journal's scope.

Response: Thank you for the comments on damage and DNA damage. We think that damage comes in various forms, and that it is hard to dilute DNA damage. There are pathways to repair damaged DNA rather than dilute it. Aging is not caused by merely mutation accumulation, and most other age-related damage forms (e.g. by-products of metabolism, damaged enzymes, misfolded proteins, epigenetic changes) could undergo either a reset or a dilution of damage to set the cells younger.

(2) It is well-known that the DNA damage is accompanied by the aging process. Several recent papers have shown that during the embryonic development, the DNA damage sensing and DNA repairing systems are rather relaxed (Kermi et al 2019, doi:10.3390/genes10050398), which allows these early cells to proliferate frequently and fast, as a consequence, DNA damages accumulate. This does not seem consistent with the damage dilution model that you proposed. Perhaps you did not mean the DNA damage, but other damages? Please clear this up.

Response: Thank you. You are right in this point - we did not mean DNA damage from here. Regarding DNA damage, embryos are rather protected by the highly expressed DNA Damage Repair genes. In the damage dilution model, malfunctioning molecules downstream of DNA (age-related splicing variants, misfolded proteins, toxic metabolic byproducts) are diluted. We think the age-related damage is a much broader term than just DNA damage itself.

(3) Some references suggest that the cell proliferation of germ cells is accompanied by apoptosis in a large portion of these germ cells, and their exiting from cell cycle into the differentiation of oocytes preludes with cell cycle arrest allowing the DNA damage sensing and repairing - This has been an established mechanism that "dilute" or remove the DNA damage from gametes. [suggested references]
Additional mechanism has also been shown very recently to reduce mutations in germ cells through transcriptional scanning: Xia et al doi: 10.1016/j.cell.2019.12.015
Thus, you might want to modify the damage dilution model by adding these mechanisms and references to clarify your point. Apparently, the DNA damage is diluted through these mechanisms.

Response: Thank you for referring to these additional mechanisms. We added more discussion on these issues. Through reproduction, DNA undergoes scanning and a high level of expression assists repair mechanisms. However, dilution involves the other forms of damage such as misfunctioning RNA, proteins, metabolites or even unknown damaged species.

(4) Embryonic stem cells and other stem cells contain inner immunity function, i. e. these cells can function like non-professional phagocytes to remove their neighboring apoptotic cells and cell debris without eliciting inflammatory response. This has been shown repeatedly in worms, flies, and mammals under EM.

Response: Thank you. We are aware of this and added it to the main text.

(5) You might want to add some examples of the AP genes in four categories that you discussed in the text.

Response: Thank you. We added some general examples in the referred categories. Particular genes like these are usually not potent, thus they are not the key genes related to certain diseases/biological processes.

(6) [discussion with author on possible titles]

3rd editorial decision 03/11/2020

Editor Comments to Author

Thank you for revising the Perspective. Before transferring it to our production team, I would like to ask you to polish the manuscript based on our edits and suggestions (see the details in the attached manuscript). These suggestions serve as the

references that intend to remind you of the minor issues that need to be clarified. Although we wish to see some emphasis on the nucleotide and epigenetic damage and their effects, and more discussion about the role of stemness in the age-related oncogenesis particularly in vivo, we will not enforce it in this paper. Also, would a single sentence title be more effective?

3rd author response to editor	03/11/2020

We would like to express our greatest appreciation for the time spending modifying this manuscript and bringing the discussion of the biology of aging to a higher level.

Please see the modifications we made in the text. We modified the manuscript based on your comments but suggest keeping the same title. Thank you.
